# Fiber-Guided Red Light-Emitting Diode Localization of an Esophageal Blind End in Long-Gap Esophageal Atresia Surgery: A Case Report

**DOI:** 10.70352/scrj.cr.25-0543

**Published:** 2026-02-20

**Authors:** Hiroki Kanamori, Tomonori Tsuchiya, Yasuhiro Kondo, Atsuki Naoe, Shunsuke Watanabe, Toshihiro Yasui, Eri Ogawa, Mikihiro Inoue

**Affiliations:** Department of Pediatric Surgery, Fujita Health University School of Medicine, Toyoake, Aichi, Japan

**Keywords:** esophageal atresia, long-gap, Gross type A, red light transillumination, Tumguide

## Abstract

**INTRODUCTION:**

Esophageal atresia (EA) Gross type A is a congenital malformation in which both the upper and lower esophagus have blind ends without a tracheoesophageal fistula. The gap between the segments is often long (long-gap EA), making intraoperative localization challenging. We report a case in which the lower esophageal blind end was readily localized using Tumguide (Otsuka Pharmaceutical Factory, Tokushima, Japan), a device that transmits biologically transparent red light from a light-emitting diode (LED) through an optical fiber.

**CASE PRESENTATION:**

The patient was a 2-month-old girl diagnosed at birth with EA Gross type A. A gastrostomy was performed shortly after birth, and esophageal lengthening with the Howard procedure was carried out over the following month. At 2 months of age, she underwent delayed primary esophageal anastomosis. At the start of surgery, the Tumguide was inserted through the gastrostomy site into a Salem Sump gastric drainage tube. The device provided clear localization of the lower esophageal blind end, minimizing the need for dissection of surrounding tissues.

**CONCLUSIONS:**

Fiber-guided red LED illumination, using Tumguide, enabled safe and straightforward intraoperative localization of the esophageal blind end in long-gap EA Gross type A. This approach minimized the need for dissection, thereby reducing the risks of injury to surrounding tissues and postoperative complications such as anastomotic stricture and leakage.

## Abbreviations

BT
: biologically transparent
EA
: esophageal atresia
ICG
: indocyanine green
LED
: light-emitting diode

## INTRODUCTION

EA Gross type A is a congenital malformation without a tracheoesophageal fistula, accounting for approximately 7% of EA cases.^1)^ It is characterized by both the upper and lower esophagus ending blindly, usually with a long gap between them, which makes primary esophageal anastomosis almost always impossible. In most cases, a gastrostomy is performed at birth, followed by esophageal lengthening procedures. These include extrathoracic esophageal elongation (Foker procedure), bougienage (Howard procedure), migration of cervical esophagostomy (Kimura procedure), and, more recently, esophageal magnetic anastomosis.^2)^

Once the upper and lower esophageal blind ends are sufficiently lengthened, a delayed primary anastomosis is attempted. However, intraoperative localization of these blind ends— particularly the lower one—remains technically challenging. Usually, both blind ends are localized using gentle compression and palpation: the upper one with an orally inserted gastric tube or Nelaton catheter and the lower one with a probe, Hegar dilator, or gastric tube inserted through a gastrostomy site. The lower blind end, however, is often thin, fragile, and susceptible to ischemia, especially if excessive dissection is required.

Facilitating the localization of the esophageal blind ends while minimizing surrounding tissue dissection can reduce the risk of postoperative complications such as anastomotic stricture and leakage. To this end, ICG fluorescence dye injection has been reported as an adjuvant,^3)^ but it has not become an established technique. Thoracoscopic repair, while increasingly popular in EA surgery, is generally unsuitable for long-gap EA, as it provides only a limited operative field and lacks tactile feedback at the tissue scale of interest.

Here, we describe a case of EA Gross type A in which the lower esophageal blind end was readily and safely identified using the Tumguide (Otsuka Pharmaceutical Factory, Tokushima, Japan), a device, introduced in October 2023, that transmits BT red light from an LED through an optical fiber. The primary intended use of this product is fluorescent guidance during nasogastric tube insertion, and the method introduced in this work constitutes an off-label use.

## CASE PRESENTATION

**Fig. 1:**
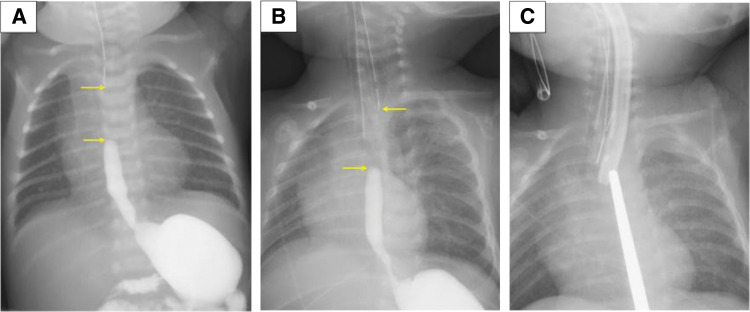
(**A**) The gap between the upper and lower esophagus at birth was 3.5 vertebral bodies. (**B**) Preoperative contrast imaging showed that the upper esophageal blind end was at a relatively high position, while the lower one reached the Th5 vertebra in its natural position. Yellow arrows indicate the blind ends of the upper and lower esophagus in (**A**) and (**B**). (**C**) The anticipated site of overlap was approximately at the Th3–Th4 vertebra by the Howard procedure.

The patient was a 2-month-old girl who had been diagnosed at birth with EA Gross type A, tetralogy of Fallot, and tracheomalacia. As her cardiac function was preserved, treatment of EA was prioritized. A gastrostomy was performed at birth, followed by esophageal lengthening with the Howard procedure. Although the gap between the upper and lower esophagus at birth was approximately 3.5 vertebral bodies, by 2 months of age the Howard procedure had reduced the gap: the upper blind end was located above the Th3–Th4 level, the lower blind end extended to Th5 (**Fig. 1B**), and the anticipated site of approximation was projected around the Th3–Th4 vertebral level (**Fig. 1C**).

**Fig. 2:**
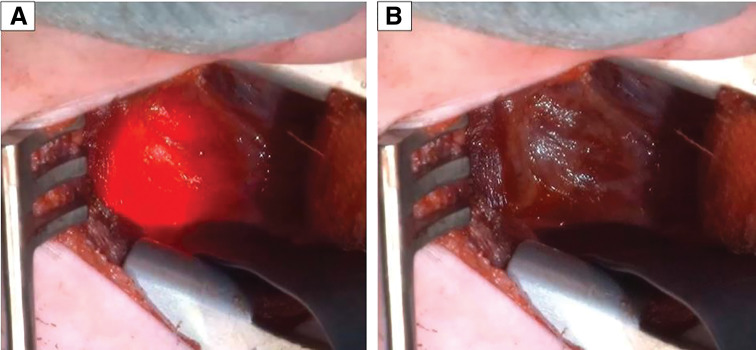
The Tumguide, a BT red LED illumination device, enabled clear and safe identification of the lower esophageal blind end; it was activated in (**A**), not in (**B**). BT, biologically transparent; LED, light-emitting diode

Based on this radiological assessment, delayed primary esophageal anastomosis was deemed feasible, and we proceeded with the operation through a right mid-axillary line incision and a fourth intercostal thoracotomy. Prior to the operation, a 14-Fr Salem Sump tube (Cardinal Health, Tokyo, Japan) was inserted through the gastrostomy site under fluoroscopic guidance, with a pediatric stylet placed inside to provide stiffness. Through the tube’s lumen, the optical fiber of the Tumguide device was advanced to its distal tip, enabling illumination at the esophageal blind end with red BT light transmitted from the device’s external LED source. We selected the Tumguide fiber with an outer diameter of 1.0 mm and a total length of 1300 mm, considering that it might be inserted together with a stylet. Once the fiber was in place, the stylet was removed, and the patient was positioned in the left lateral decubitus position. After confirming that the tube position had not changed, the surgery was initiated. Through an extrapleural approach to the posterior mediastinum, the azygos vein was identified. The Tumguide light was activated, and the BT red LED illumination enabled clear visualization of the blind end (**Fig. 2**), allowing safe dissection of the surrounding connective tissue and encirclement of the esophagus with minimal manipulation. A branch of the vagus nerve was observed crossing anteriorly to the esophagus, so we continued the dissection carefully and repositioned the lower esophagus posterior to the vagus nerve without injury.

Subsequently, we proceeded to explore the upper esophageal blind end. When pressure was applied to an orally inserted Nelaton catheter, its tip had not yet entered the mediastinum. Traction strings were applied to the upper esophageal pouch, and after gentle dissection of surrounding tissues, the catheter, pushing the pouch, was guided into the mediastinum. During this step, multiple episodes of oxygen desaturation and poor ventilation occurred, prompting a temporary interruption of the procedure. These adverse events were presumed to result from airway obstruction due to compression of the membranous portion of the trachea during manipulation, with tracheomalacia considered a contributing factor. Despite these difficulties, the gap between the upper and lower esophagus became sufficiently short to allow an anastomosis, and an end-to-end esophageal anastomosis was performed with a total of 8 stitches. Although the lower esophagus appeared slightly pale after anastomosis, the mucosa remained viable in color. Considering the high anastomotic position, we inserted a nasal transanastomotic tube and managed the patient under deep sedation postoperatively for 7 days. On POD 10, the esophagography showed no evidence of anastomotic stricture or leakage. There was no sign of hiatal hernia, but severe gastroesophageal reflux was noted, likely due to upward displacement of the lower esophagus for the anastomosis. Since no leakage was detected, gastrostomy and enteral feeding were started. Gastroesophageal reflux was successfully managed with medical therapy, and the patient was discharged without additional complications.

## DISCUSSION

This report describes a case of long-gap Gross type A EA in which Tumguide fiber-guided red LED illumination enabled straightforward and safe localization of the lower esophageal blind end during delayed primary anastomosis.

The management of long-gap EA remains a significant surgical challenge and is associated with a relatively high rate of postoperative complications. Both the American Pediatric Surgical Association and the European Reference Network for Rare Inherited Congenital Anomalies recommend delayed primary anastomosis when the gap between the esophageal ends is too extensive to permit immediate anastomosis.^4,5)^ Esophageal lengthening plays a crucial role in this approach, but leakage and stricture rates remain high, reaching 30% and 60%, respectively.^6,7)^ Gentle handling and preservation of blood supply are critical, and unnecessary dissection should be avoided. In Gross type A EA, however, the absence of a tracheoesophageal fistula makes intraoperative localization of the blind ends difficult, often necessitating extensive dissection and risking injury.

To address this issue, we utilized the Tumguide light source and fiber, a BT red LED system, inserted through a gastric tube to facilitate intraoperative identification of the esophageal blind end. The device, manufactured by Otsuka Pharmaceutical Factory, has primarily been used to confirm correct positioning of gastric tubes, where the transmitted red light allows visualization of the tube tip position through the abdominal wall. In recent years, reports of its use in children have increased, with Satake et al.^8)^ reporting a sensitivity for tube tip localization of 99% compared with radiographic confirmation.

**Fig. 3:**
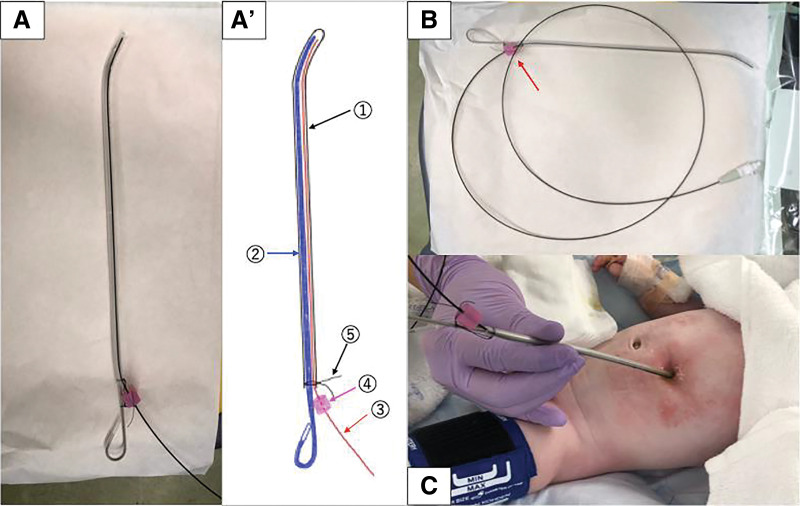
(**A**, **A**′, **B**) A 14-Fr Salem Sump tube (①) was cut at its midpoint, and a pediatric intubation stylet (②)—its tip bent slightly by us— was inserted to facilitate fluoroscopic guidance of the tube into the lower esophagus. The Tumguide fiber (③) was also inserted into this assembly, and its position was secured with surgical thread (④) in addition to the device’s stopper (④). (**C**) The custom-made tube assembly was inserted through the gastrostomy site under fluoroscopic guidance and advanced into the lower esophagus, after which the stylet was removed.

The Tumguide optical fiber is relatively rigid, and direct exposure of the bare tip to tissue is contraindicated due to the risk of perforation. For safety, this device has a stopper to fix the fiber in place, but because the system is obscured by surgical drapes during the procedure, we devised a method to further ensure safe usage. Specifically, we modified a 14-Fr Salem Sump tube by cutting it midway and inserting a pediatric intubation stylet, which we had bent slightly at the tip to facilitate fluoroscopic guidance of the tube. The Tumguide fiber was also inserted into this assembly, and the tip position was adjusted and secured with surgical thread in addition to using the device’s stopper (**Fig. 3**). To our knowledge, this represents the first reported application of Tumguide for intraoperative localization of the lower esophageal blind end.

While our custom-adjusted Tumguide system enabled easy and accurate identification of the lower esophagus, it might have been even more beneficial for localizing the upper esophageal blind end, which had not yet reached the mediastinum. We did not attempt oral insertion of the device into the upper esophagus due to product specifications and the need to maintain smooth surgical flow, but we believe this approach could have further facilitated the procedure and improved safety. In future cases, the decision to insert the Tumguide into the upper esophagus, the lower esophagus, or both should be considered on a case-by-case basis, and its use from both directions may offer added safety and efficiency in identifying the blind ends.

## CONCLUSIONS

In the operation for esophageal anastomosis of long-gap EA, particularly Gross type A where no tracheoesophageal fistula is present, identifying the upper and lower esophagus can be technically challenging. In this case, the use of the Tumguide, a BT red LED illumination device, enabled clear and safe identification of the lower esophageal blind end. This approach minimized the need for extensive dissection, thereby reducing the risk of intraoperative injury and postoperative complications such as anastomotic stricture and leakage. We believe the Tumguide can serve as a useful adjunct in the surgical management of complex long-gap EA cases.

## DECLARATIONS

### Availability of data and materials

Data sharing is not applicable to this article, as no datasets were generated or analyzed during the current study.
